# Identification of Somatic Genetic Alterations Using Whole-Exome Sequencing of Uterine Leiomyosarcoma Tumors

**DOI:** 10.3389/fonc.2021.687899

**Published:** 2021-06-11

**Authors:** Lihua Chen, Jiajia Li, Xiaohua Wu, Zhong Zheng

**Affiliations:** ^1^ Department of Gynecologic Oncology, Fudan University Shanghai Cancer Center, Shanghai, China; ^2^ Department of Oncology, Shanghai Medical College, Fudan University, Shanghai, China

**Keywords:** exome sequencing, uterine leiomyosarcoma, *SHARPIN*, TP53, ULMS

## Abstract

**Background:**

The genomic abnormalities associated with uterine leiomyosarcoma (uLMS) have not been fully elucidated to date.

**Objective:**

To understand the pathogenesis of uLMS and to identify driver mutations and potential therapeutic targets in uLMS.

**Methods:**

Three matched tumor-constitutional DNA pairs from patients with recurrent uLMS were subjected to whole-exome capture and next-generation sequencing. The role of the selected gene *SHARPIN* in uLMS was analyzed by the CCK-8 assay and colony formation assay after specific siRNA knockdown.

**Results:**

We identified four genes with somatic SNVs, namely, SLC39A7, GPR19, ZNF717, and TP53, that could be driver mutations. We observed that 30.7% (4/13) of patients with uLMS had TP53 mutations as analyzed by direct sequencing. Analysis of somatic copy number variants (CNVs) showed regions of chromosomal gain at 1q21-23, 19p13, 17q21, and 17q25, whereas regions of chromosomal loss were observed at 2q35, 2q37, 1p36, 10q26, 6p22, 8q24, 11p15, 11q12, and 9p21. The *SHARPIN* gene was amplified in two patients and mutated in another (*SHARPIN*: NM_030974: exon2: c.G264C, p.E88D). Amplification of the *SHARPIN* gene was associated with shorter PFS and OS in soft tissue sarcoma, as shown by TCGA database analysis. Knockdown of *SHARPIN* expression was observed to decrease cell growth and colony formation in uterine sarcoma cell lines.

**Conclusions:**

Exome sequencing revealed mutational heterogeneity of uLMS. The *SHARPIN* gene was amplified in uLMS and could be a candidate oncogene.

## Introduction

Uterine leiomyosarcoma (uLMS) is a rare but aggressive malignancy. uLMS accounts for only 1–3% of all uterine malignancies, exhibiting an annual incidence rate of 0.4–0.9/100,000 women, but it is the most common type of uterine sarcoma (1). uLMS is a highly malignant disease, with 5-year survival rates averaging approximately 40%. Although many patients are diagnosed with early-stage disease, the recurrence rate, even among patients with uterine confined disease (FIGO stage I), is greater than 50% (2). For these cases of recurrent uLMS, no curative treatment has been identified to date. Patients with single recurrent disease may be considered for secondary cytoreductive surgery (3). Chemotherapy is also considered to be effective for the treatment of advanced or recurrent uLMS. However, the response rates of uLMS to current cytotoxic agents are disappointing, with partial response rates varying from 0% to 33% and complete response rates varying from 0% to 8% (4). Targeted therapy and immunotherapy have also been heavily studied. Olaratumab and pazopanib are two new targeted-therapy drugs that were recently approved by the Food and Drug Administration (FDA) for treating advanced soft-tissue sarcomas, including uLMS. The objective response rate of pazopanib in LMS is only 6%, with no significant improvement in overall survival (OS), as shown in the randomized, placebo-controlled PALETTE trial (5). Olaratumab is a human antibody directed against PDGF-α. In a randomized phase II study, Olaratumab plus doxorubicin exhibited a response rate of 18.2% and improved PFS and OS compared with those in a control group (6). However, the later phase III study ANNOUNCE showed that the combination of Olaratumab and doxorubicin produced no significant difference in median overall survival compared with doxorubicin alone (7). Therefore, the effects of these drugs are limited. The development of more effective targeted therapies for uLMS warrants further research.

The genomic abnormalities associated with uLMS have not been fully elucidated to date, and no targeted therapy has been established for this cancer. No single driving mutation has been identified for uLMS. Most tumors exhibit multiple somatic chromosomal abnormalities. Genetic profiling is investigational in LMS but could eventually elucidate treatment targets (8). Uterine LMS exhibits multiple and varied genetic aberrations and very complex, often aneuploid or polyploid, karyotypes (9). This heterogeneity complicates the identification of driver mutations and therapeutic targets. To understand the pathogenesis of uLMS recurrence and identify the driver mutations and potential therapeutic targets in uLMS, we performed whole-exome sequencing on three paired tumors and normal samples from patients with uLMS. We identified several potential driving mutations, and amplification of the *SHARPIN* (Shank-associated RH domain-interacting protein) gene may be involved in uLMS progression.

## Methods

### Patients

The Institutional Review Board of Fudan University Shanghai Cancer Center approved this study, and all participants, or parents of participants, provided written informed consent before samples were collected. We collected tumor tissue and tumor-adjacent normal tissue from three patients with uLMS for exome sequencing, and we collected samples from another 10 patients for TP53 sequencing. The study was conducted in accordance with the Declaration of Helsinki and its amendments and with Good Clinical Practice guidelines.

### DNA Extraction and Sequencing

Genomic DNA was extracted from constitutional or tumor tissues using a QIAmp DNA Minikit (Qiagen, Valencia, California). Sample libraries were prepared using the TruSeq^®^ DNA LT/HT Sample Prep Kit (Illumina, Inc., San Diego, CA) and TruSeq^®^ Exome Enrichment Kit (Illumina, Inc.) following the manufacturer’s instructions. Captured libraries were sequenced with HiSeq 2500 (Illumina Inc.) at RiboBio Co., Ltd. (Guangzhou, China).

### Somatic Variant Analysis

Sequence reads were mapped to the reference genome (hg19) using the BWA program (version 0.7.12) (10). Single-nucleotide polymorphisms (SNPs) were identified by SAMTOOLS software and annotated by ANNOVAR (version) (11). Somatic mutations were defined as mutations that were identified in tumor DNA but were absent from the normal-tissue DNA. A web-based ANNOVAR (11) and the cancer-related analysis of variants toolkit (CRAVAT 4) (12) were employed to eliminate false-positive findings and identify the driver somatic mutations. Somatic copy number alterations (SCNAs) were detected as deviations from the adjusted log-ratio of sequence coverage depth within a tumor-normal pair as reported previously (13, 14). When the adjusted ratio value was > 0.20, the SCNAs were regarded as amplifications, and when the ratio value was < - 0.10 the SCNAs were regarded as deletions. The identified SCNAs were subjected to gene set enrichment analysis using WebGestalt (WEB-based Gene SeT AnaLysis Toolkit) (15) for GO pathway analysis and chromosomal location.

### TP53 Direct Sequencing

DNA sequencing of the purified DNA products was performed by GENEWIZ, Inc. (Suzhou, China) using BigDye version 3.1 in ABI2720 (Applied Biosystems CA, USA.) for PCR. The sequencing reactions were subsequently run on an Applied Biosystems 3730XL Analyzer. The primers for the TP53 direct sequence are shown in **Table S1**.

### Cell Culture and *SHARPIN* siRNA Transfection

Human uterine sarcoma cell lines SK-UT-1 and MES-SA were obtained from ATCC. MES-SA was cultured in Gibco™ DMEM (Carlsbad, CA), and SK-UT-1 was cultured in Gibco™ MEM supplemented with 10% FBS (Grand Island, NY, USA). *SHARPIN* siRNA (target sequence 5’-CCCTGAGTGTTCAGCTTCA-3’) and negative-control siRNA (target sequence 5’-GGCTCTAGAAAAGCCTATGC-3’) were transfected into cells (5 µg siRNA, ratio 1:1 duplexes) using ExFect2000 Transfection reagent (Vazyme, Nanjing, China) for 72 h according to the manufacturer’s instructions and were subsequently collected for Cell Counting Kit-8 (CCK-8), Western blot (WB) and colony formation assays.

### Cell Counting Kit-8 (CCK-8) Assay

After transfection for 72 h, cells were plated in 96-well microplates and cultured with growth medium for 24, 48 and 72 h. Cell proliferation was measured after incubation with Cell Counting Kit-8 (Multisciences Biotech Co., Hangzhou, China) reagent for an additional 1 h at 37°C, and optical density at 450 nm/650 nm was measured for each well.

### Western Blot Analysis

Western blot analysis was performed as previously described (16). Briefly, cell lysates were separated by SDS-PAGE and transferred to a PVDF membrane (Millipore, Billerica, MA, USA). The blot was subsequently probed with anti-*SHARPIN* (abs134288, ABSIN, Beijing, China) at a dilution of 1:500 or anti-GAPDH (Multisciences Biotech Co., Hangzhou, China) at a dilution of 1:5000, followed by incubation with a horseradish peroxidase-conjugated secondary antibody. The signal was detected using enhanced chemiluminescence (Millipore, Billerica, MA, USA). The expression level was quantified using the ImageJ program (NIH).

### Colony Formation Assay

Cell lines were seeded in six-well plates (3×10^2^/well) and cultured until colonies were visible. Cells were fixed with chilled methanol for 10 min, stained with 0.5% crystal violet in 25% methanol for 20 min, and photographed after overnight drying.

### Statistical Analysis

Statistical analyses of data were performed by using Student’s t-test or one-way analysis of variance for two-group comparisons. Data that failed the test for normal distribution or homogeneous variance were analyzed using the Mann-Whitney U test. The statistical software SPSS version 24.0 (IBM Corp. Armonk, NY, USA) was utilized for analysis. P-values < 0.05 were considered to be significant.

## Results

We performed exome sequencing of 3 uterine leiomyosarcomas and their matched normal controls. The tumor tissue was obtained during the second cytoreductive surgery, and the characteristics of the patients are shown in **Table 1**. Exome sequencing generated an average of 13.9 gigabases of raw data per sample. The total number of reads per sample ranged from approximately 62,858,796 to 100,704,888. The overall average coverage ranged from 56x to 96x, with 80.1-85.1% of reads covered a minimum of 20 times (**Supplementary Table S2** and **Figure S1**).

**Table 1:** The characteristics of the patients and the tumor tissue was obtained during the second cytoreductive surgery.

	P1	P2	P3
**Age**	40s	60s	50s
**Stage**	IB	IB	IB
**treatment^#^**	Surgical+chemotherapy (ADM+IFO)	Surgical+chemotherapy (ADM+IFO)	Surgical+chemotherapy (PAC+L-OXP)
**differentiation**	High grade	Low grade	High grade
**progression-free survival (month)**	24	16	9
**Overall survival (month)**	43 (dead)	63.8 (survial with disease)	25 (dead)

^#^ADM, Adriamycin; IFO, Ifosfamide; PAC, Paclltaxel; L-OXP, Oxaliplatin.

### Somatic Mutations

**Figure 1:**
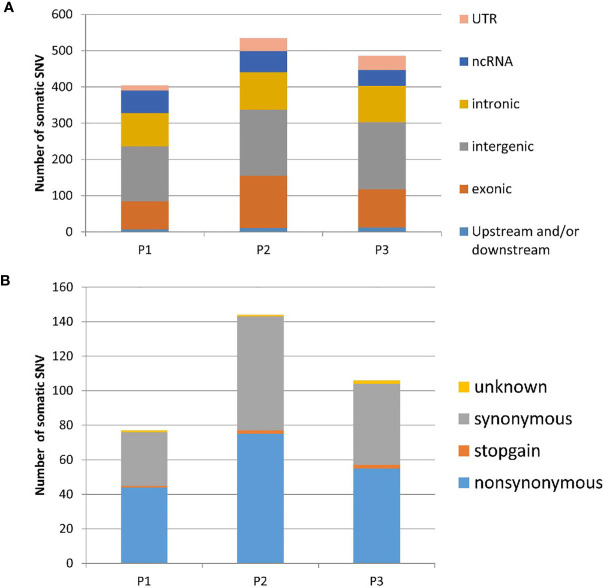
Distribution of somatic SNVs in three uLMS patients identified by exomic sequence **(A)**. The component of exonic SNV annotated by ANNOVAR **(B)**. the component of exonic somatic SNV annotated by ANNOVAR. UTR, untranslated region; ncRNA, non-coding RNA; P1, Patients 1; P2, Patients 2; P3, Patients 3; SNV, single nucleotide variants.

Exome sequencing identified 155459-184924 single nucleotide variants (SNVs) in each tumor or control sample (**Table S3**). Nearly 5% of the SNVs had not been previously reported in dbSNP, the 1000 Genomes Project or ESP6500. The SNVs consisted of 51.5% synonymous, 48.02% nonsynonymous and 0.44% stop gain and loss mutations. The SNVs were observed to be distributed in various genomic regions, including downstream regions (1.05%), exonic regions (11.7%), splicing regions (0.2%), intergenic regions (31.51%), intronic regions (37.44%), ncRNA-encoding regions (5.8%), upstream regions (1.56%), 5’-untranslated regions (1.66%) and 3’-untranslated regions (9.97%). Our exome sequencing identified 1420 somatic mutations in all three patients, 174 of which were determined to be exonic nonsynonymous mutations (**Figures 1A, B**). To identify disease-related mutations, we first employed the Cancer-Related Analysis of Variants Toolkit (CRAVAT) for genomic variant interpretation. When the *P-*values of CHASM (Cancer-specific High-throughput Annotation of Somatic Mutations) and VEST (Variant Effect Scoring Tool) were both set at < 0.05, four genes with mutations were determined to be significant: SLC39A7, GPR19, ZNF717, and TP53 (**Table 2**). Web-based ANNOVAR (wANNOVAR) was also used to annotate functional consequences of genetic variation from our high-throughput sequencing data. This tool employs several steps to identify a subset of potentially deleterious variants/genes *via* the web program. In total, 13 variants remained after filtration (**Table S4**), and these genes were then submitted automatically as input into the Phenolyzer together with the term ‘uterine leiomyosarcoma’ by wANNOVAR. The visualized network drawn by Phenolyzer is shown in **Figure S2**. The TP53 gene was ranked at the top based on the resulting network. All four gene variants identified by CRAVAT were included among the 13 variants identified by wANNOVAR. Next, we confirmed the mutation of TP53 by direct sequencing in these patients and in another 10 uLMS patients. In total, we observed that 30.7% (4/13) of patients had TP53 mutations.

**Table 2:** Four genes with disease-related mutations were determined to be significant: SLC39A7, GPR19, ZNF717, and TP53 by using the Cancer-Related Analysis of Variants Toolkit (CRAVAT) for genomic variant interpretation.

HUGO symbol	SLC39A7	GPR19	ZNF717	TP53
**Chrom**	**chr6**	**chr12**	**chr3**	**chr17**
**Position**	33171585	12815066	75790516	7577539
**Strand**	+	+	+	+
**Ref. base(s)**	G	G	T	G
**Alt. base(s)**	C	C	A	A
**Sample ID**	P1	P2	P3	P3
**Sequence ontology**	MS	MS	MS	MS
**Protein sequence change**	E469Q	S106C	H63L	R248W
**CHASM cancer driver p-value (missense)**	0.0064	0.0193	0.0247	0.0016
**CHASM cancer driver FDR (missense)**	0.2	0.4	0.4	0.1
**VEST pathogenicity p-value (non-silent)**	0.01875577	0.00651641	0.025987	0.00672533
**VEST pathogenicity FDR (non-silent)**	0.25	0.2	0.3	0.2
**dbSNP**			rs201105907	rs121912651
**1000 Genomes allele frequency**	0	0	0	0
**COSMIC ID**	COSM4852625		COSM4594535	COSM10656

And the P-values of CHASM (Cancer-specific High-throughput Annotation of Somatic Mutations) and VEST (Variant Effect Scoring Tool) were both set at < 0.05.

### Somatic Indels and Copy Number Alterations (CNAs)

A total of 6,252-8,008 indels were detected for each sample. The annotation of these indels by ANNOVAR is shown in the supplementary data (**Table S5**). Nearly half of the indels were located in introns, followed by intergenes and 3’-UTRs. Somatic CNAs (SCNAs) were detected as deviations from the log-ratio of sequence coverage depth within a tumor–normal pair (14) (adjusted log ratio > 0.25), deletions (adjusted log ratio < -0.10), or neutral (between -0.1 and 0.25). There were 8097 loci (5937 genes), 2340 loci (1932 genes), and 5392 loci (4334 genes) identified in each case. Gene set enrichment analysis of chromosomal location showed SCNA locations among different cases (**Figure S3**) and GO pathways (**Figure S4**). The GO pathways with false discovery rates (FDRs) < 0.05 are eicosanoids, fatty acids, complex I biogenesis, cell cycle checkpoints, homology-directed repair, transcriptional regulation by RUNX1, pre-NOTCH expression, chromosome maintenance, mitotic prophase, CENPA-containing nucleosomes, reproduction, and condensation of prophase chromosomes. Analyses of somatic CNAs showed that regions of chromosomal gain are 1q21-23, 19p13, 17q21, and 17q25, whereas regions of chromosomal loss are 2q35, 2q37, 1p36, 10q26, 6p22, 8q24, 11p15, 11q12, and 9p21. Among these regions, alterations in 1q21, 19p13 and 2q35 were observed in two patients.

### SHARPIN

**Figure 2:**
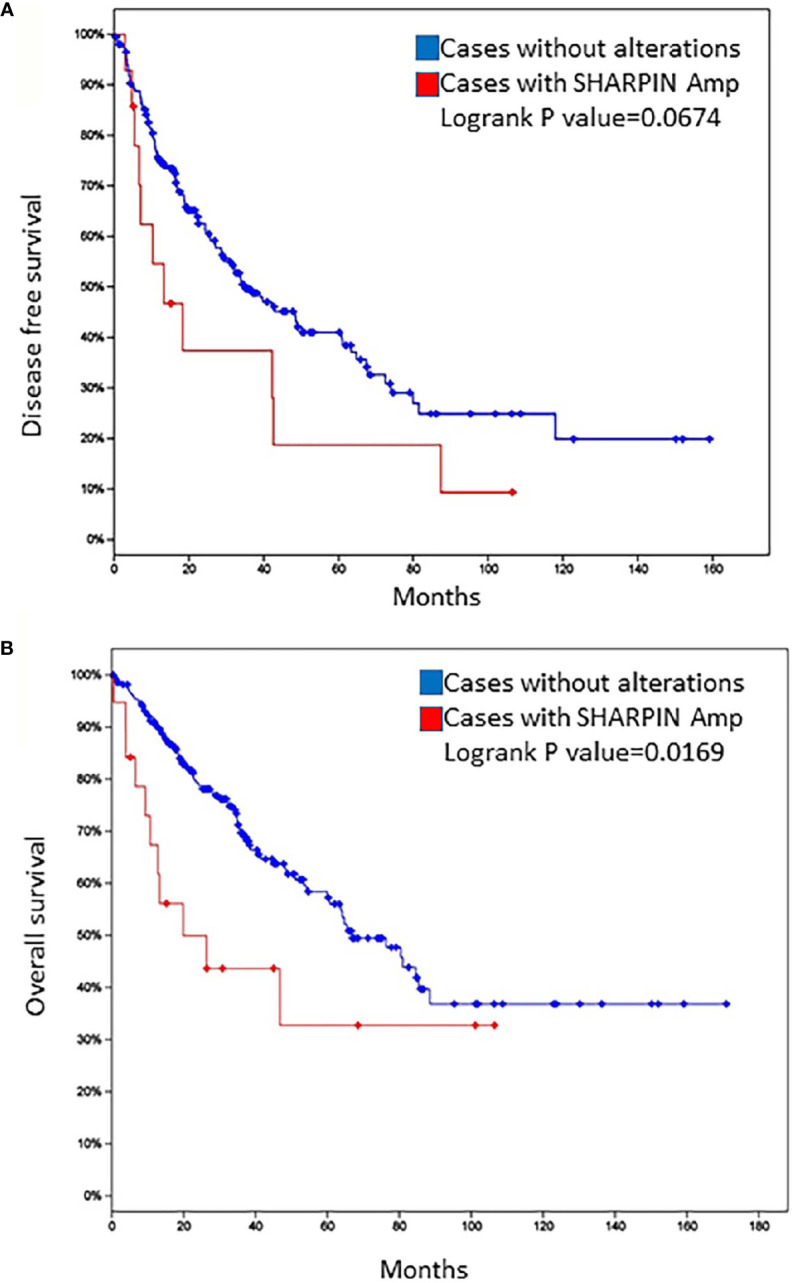
Disease-free survival **(A)** and overall survival **(B)** of patients with sarcoma in the TCGA database according to the status of *SHARPIN* gene amplification.

**Figure 3:**
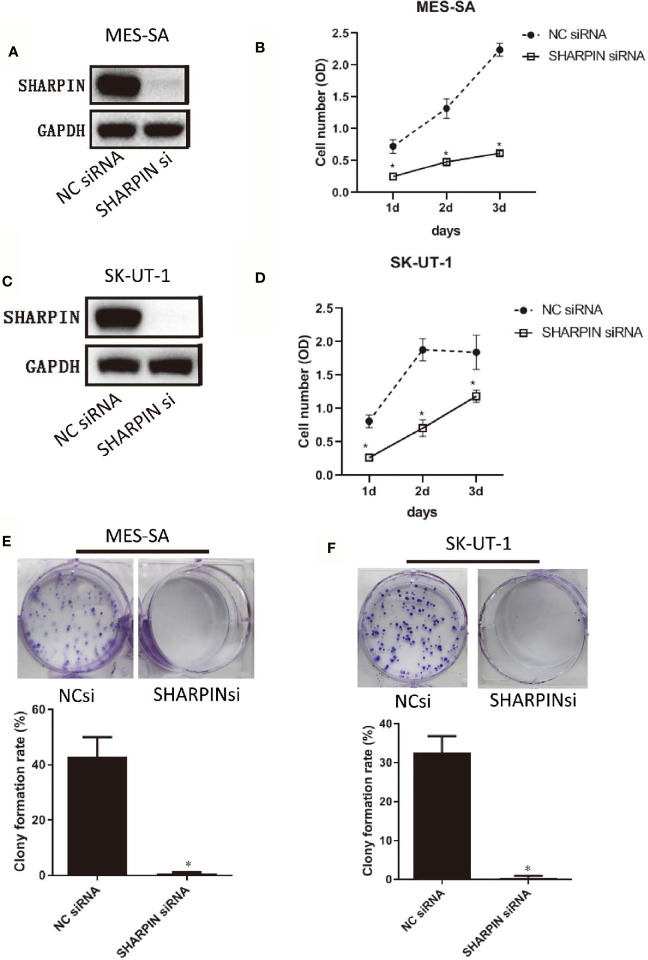
Knockdown of *SHARPIN* expression decreased uterine sarcoma cell proliferation and colony formation. **(A)**, Western blot analysis of *SHARPIN* expression in MES-SA cells transfected with negative control (NC) siNRA or *SHARPIN* siRNA for 72 h. **(B)**, MES-SA cells were transfected with *SHARPIN* or NC siRNA for 72 h and plated in 96-well plates. Cell proliferation was analyzed daily by the CCK-8 assay during the following three days. Data were obtained from three independent experiments in triplicate. **(C)**, Western blot analysis of *SHARPIN* expression in SK-UT-1 cells transfected with NC siNRA or *SHARPIN* siRNA for 72 h. **(D)**, SK-UT-1 cells were transfected with *SHARPIN* or NC siRNA for 72 h and plated in 96-well plates. Cell proliferation was analyzed daily by the CCK-8 assay during the following three days. Data were obtained from three independent experiments performed in triplicate. *p < 0.05 vs. NC shRNA; n = 3. **(E, F)**, Colony formation assay of MES-SA **(E)** and SK-UT-1 **(F)** cells after transfection with *SHARPIN* or NC siRNA for 72 h. *p < 0.05 vs. NC shRNA; n = 3. Full-length blots from **Figures 3A, C** are shown in **Figure S5**.

When examining all the genes of interest, one gene, *SHARPIN*, was notable. The *SHARPIN* gene was amplified in patients 1 (adjusted_log_ratio is 0.547) and 3 (adjusted_log_ratio is 0.263) and mutated (*SHARPIN :* NM_030974:exon2:c.G264C:p. E88D) in patient 2. This gene is one of the 13 variants identified by wANNOVAR. By searching the TCGA sarcoma database (http://www.cbioportal.org.TCGAcell2017), we observed that the *SHARPIN* gene was amplified in 5% of sarcomas and was associated with shorter DFS and OS (**Figure 2**). To investigate the role of the *SHARPIN* gene in the carcinogenesis of uLMS, we knocked down *SHARPIN* expression in two uLMS cell lines (**Figures 3A, C** and **S5**) and compared cell proliferation with the CCK-8 assay and the colony formation assay. Silencing *SHARPIN* expression decreased cell growth in both uLMS cell lines (**Figures 3B, D**) and decreased uLMS colony formation (**Figures 3E, F**).

## Discussion

In this study, we employed exome sequencing to examine somatic variation in 3 uLMS. We identified four genes with somatic SNVs, namely, SLC39A7, GPR19, ZNF717, and TP53, that may have clinical relevance. Analyses of somatic copy number variants (CNVs) showed that regions of chromosomal gain are 1q21-23, 19p13, 17q21, and 17q25, whereas regions of chromosomal loss are 2q35, 2q37, 1p36, 10q26, 6p22, 8q24, 11p15, 11q12, and 9p21. Next, we searched the TCGA database for progression-related gene variation from our identified somatic CNVs and SNVs. We found that *SHARPIN* amplification was associated with poor progression in LMS. In our study, the SHARPIN gene was amplified in patients 1 and 3 (high grade uLMS) and mutated (SHARPIN:exon2:c.G264C:p. E88D) in patient 2 (low grade uLMS). The overall survival of patient 2 was much longer than that of patient 1 and 3. Based on these findings, we hypothesized that SHARPIN might be associated with the prognosis of uLMS. Knockdown of *SHARPIN* expression decreased cell growth and cell colony formation in uterine sarcoma cell lines. This finding indicated the oncogenic role of *SHARPIN* in uLMS and demonstrated that *SHARPIN* could be utilized as a potential therapeutic target in uLMS.

*SHARPIN* is a major component of the E3 ubiquitin-protein ligase complex, the linear ubiquitin chain assembly complex (LUBAC), and it plays essential roles in many processes, including normal tissue development, inflammation, homeostasis and carcinogenesis. Recent studies have shown that *SHARPIN* is frequently upregulated in multiple human cancer types, including ovarian, prostate (17), and breast cancers (18), hepatocellular carcinoma (19) and melanoma (20). *SHARPIN* promotes cancer cell survival, growth, invasion and metastasis (17–19). However, *SHARPIN* inhibits esophageal squamous cell carcinoma progression (21). Diane Ojo et al. reported that the *SHARPIN* gene is frequently amplified in approximately 20% of breast cancers, *SHARPIN* gene copy number amplification occurred in 403 tumors among 1980 breast cancer cases in the Curtis subdataset within the cBioPortal database, and the amplification was modestly associated with a decrease in OS in breast cancer patients (22). Our results demonstrated that two out of three uLMS patients had *SHARPIN* gene amplification. We also found that nearly 5% of LMS tumors had *SHARPIN* gene copy number amplification in the cBioPortal database and that this amplification was associated with a decrease in OS and PFS. Furthermore, our cell function study confirmed that *SHARPIN* plays an important role in uterine sarcoma cell proliferation and cell colony formation. To the best of our knowledge, the present study is the first to report the oncogenic function of *SHARPIN* in uLMS. We also first found a nonsynonymous somatic mutation of the *SHARPIN* gene (exon 2:c.G264C:p. E88D), which was predicted to be deleterious by SIFT and Polyphen2. All of these findings indicate that further study is warranted to determine whether *SHARPIN* could be utilized as a potential therapeutic target in uLMS.

It has been reported that sarcomas have low mutational burdens compared with other tumors in TCGA studies (23). S. Murraya et al. performed mutational analysis of 20 exons from 9 tyrosine kinase genes and showed a low frequency of somatic mutations in uterine sarcomas (24). Recently, a study employing whole-exome and transcriptome sequencing of LMS showed that the median somatic mutation rate was 3.09 (range, 1.05–14.76) per megabase (Mb) of the target sequence (25). A recent study of 216 patients with uLMS from the cBioPortal and AACR-GENIE databases showed that the vast majority of patients (81%) carried at least one mutation in either TP53, RB1, ATRX or PTEN, while 80 patients with uLMS from the cBioPortal did not have SHARPIN amplification, possibly due to ethnic differences (26). The mutation of TP53 was also observed in our study. TP53 was the most commonly mutated gene, with 32% of the tumors harboring mutations. Alterations in TP53 have been previously implicated in leiomyosarcomas and suggested to play a role in leiomyosarcoma pathogenesis. TP53, in particular, has been frequently reported to harbor somatic mutations in leiomyosarcomas, and its presence has been proposed as a distinguishing factor between benign leiomyomas and malignant leiomyosarcomas. The spectrum of TP53 missense mutations is very broad, with more than 4,000 different alterations reported (27). Several studies suggested that recurrent gene mutations are infrequent in LMS. TP53 gene mutations were the most common abnormalities identified in 37% of the cases (28). This finding is in accordance with the 39% rate of TP53 mutations reported in a study by Ito et al (29). Our study also detected a small number of somatic SNVs and showed heterogeneity of the somatic mutation, as there is no common somatic SNV between each sample. We also identified TP53 as the driver of somatic mutations by wANNOVAR, and the rate of TP53 mutation (30.7%) was similar to that observed in a previous study.

The other three somatic mutated genes (SLC39A7, GPR19, ZNF717) that we identified were also reported to be involved in carcinogenesis. SLC39A7, also known as ZIP7, is activated by phosphorylation-mediated zinc release from intracellular stores, drives major pathways, such as MAPK, mTOR and PI3K-AKT (30), that are involved in cell survival and proliferation, and it was reported to be overactivated in breast cancer (31) and cervical cancer (32). G protein-coupled receptor 19 (GPR19) is frequently overexpressed in tissue samples obtained from patients with small cell lung cancer and supports G (2)-M cell cycle progression (33). Deleterious mutations in the transcription factor ZN717 were also identified by whole-exome or genomic sequencing in several types of cancer, such as colorectal cancer (34), hepatitis B virus-induced hepatocellular carcinoma (35) and gastric cancer (36). All of this evidence indicates that the somatic SNV identified in this study warrants further study and may provide an enhanced understanding of uLMS carcinogenesis.

A previous study showed that DNA copy number alterations are frequently observed in extrauterine LMS (28). Several studies have shown that the most frequent reported regions of chromosomal loss are 1p12, 2p, 13q, 10q, and 16q, and the most frequently reported gains are in chromosome arms 17p, 15q, 8q, and 5p in LMS (37, 38). A recent study employing targeted exome sequencing showed that the most common chromosomal losses were observed in 10q23 (PTEN), 13q14 (RB1), 16q22 (CDH1), and 17p13 (TP53) (23, 28). Our study showed different regions of chromosomal loss (2q35, 2q37, 1p36, 10q26, 6p22, 8q24, 11p15, 11q12, 9p21) and gain (1q21-23, 19p13, 17q21, 17q25), which may be observed because uLMS may have differences in genetic variation due to its heterogeneity.

This study had several key limitations. There were limited number of patients in the study. However, considering the rigor of the sequencing data, we utilized patient paired samples. And by direct sequencing of TP53, we have partly verified the credibility of our data. Because sequencing was done on recurrent tumors with unavailable primary tumor, and received different primary therapies, it is uncertain if primary chemotherapy can impact genetic makeup of recurrent tumor. Nevertheless, one of our aim in this study was to identify therapeutic targets for relapsed uLMS patients undergoing multiline therapy by exome sequencing. Genetic alterations induced by chemotherapy may also can be used for therapeutic targets.

In conclusion, exomic sequencing of uLMS samples was employed to identify four genes (SLC39A7, GPR19, ZNF717, and TP53) with somatic single nucleotide variants that could be driver mutations of uLMS, and amplification of the *SHARPIN* gene was observed. An *in vitro* study showed the oncogenic function of the *SHARPIN* gene in uterine leiomyosarcoma.

## Data Availability Statement

According to national legislation/guidelines, specifically the Administrative Regulations of the People’s Republic of China on Human Genetic Resources (http://www.gov.cn/zhengce/content/2019-06/10/content_5398829.htm, http://english.www.gov.cn/policies/latest_releases/2019/06/10/content_281476708945462.htm), no additional raw data is available at this time. Data of this project can be accessed after an approval application to the China National Genebank (CNGB, https://db.cngb.org/cnsa/). Please refer to https://db.cngb.org/, or email: CNGBdb@cngb.org for detailed application guidance.

## Ethics Statement

Ethical review and approval was obtained for the study on human participants in accordance with the local legislation and institutional requirements. Written informed consent for participation was obtained for this study in accordance with the national legislation and the institutional requirements.

## Author Contributions

ZZ contributed to the conception of the study; LC performed the experiment; JL contributed significantly to analysis and manuscript preparation; LC and ZZ performed the data analyses and wrote the manuscript; XW helped perform the analysis with constructive discussions. All authors contributed to the article and approved the submitted version.

## Funding

This work was supported by the Chinese Society of Clinical Oncology (CSCO)-Rhoche oncologic research foundation (Y-2019Roche-077), Chinese Society of Clinical Oncology (CSCO)-BMS oncologic research foundation (Y-BMS2019-018) and Pandeng Foundation of China National Cancer Center (NCC201809B032). The funding bodies played no role in the design of the study; collection, analysis and interpretation of data; and writing of the manuscript.

## Conflict of Interest

The authors declare that the research was conducted in the absence of any commercial or financial relationships that could be construed as a potential conflict of interest.
