# Supplementary material for: Identification of Somatic Genetic Alterations Using Whole-Exome Sequencing of Uterine Leiomyosarcoma Tumors
Source: Front Oncol. 2021 Jun 11;11:687899. doi: 10.3389/fonc.2021.687899 (PMC8226214; doi:10.3389/fonc.2021.687899)
Supplement: Supplementary file 1 [file DataSheet_1.docx]

**Supplementary figure legends**

Fig. S1. Summarize all the reads coverage and statistical results of variation at sites of each chromosome. The outer circle represents each chromosome, arranged in a clockwise direction, and the position of the centromere is indicated by a red line; the gray rectangle bar in the outer circle shows the coverage of the chromosome reads; the dark green point between the two circles is the deletion site, and the light green point is the insertion site. The other variation types were homozygous SNP (orange rectangle), heterozygous SNP (yellow rectangle), terminator deletion (black dot), terminator acquisition (blue dot), synonymous mutation (red), missense mutation (purple).

Fig. S2. The visualized network build by wANNOVAR analysis of somatic SNV.

Fig. S3. Gene set enrichment analysis of somatic copy number alterations (CNAs) identified by exomic sequence. Chromosomal location of somatic CNAs in P1 (a), P2 (b) and P3 (c).

Fig. S4 GO pathways of somatic CNAs in P1 (a), P2 (b) and P3 (c).

Fig. S5. Full-length blots of Fig4, A Full-length blots of fig4a SHARPIN, B Full-length blots of fig4a GAPDH, C Full-length blots of fig4c SHARPIN, D Full-length blots of fig4c GAPDH.

**Fig.S1**

**
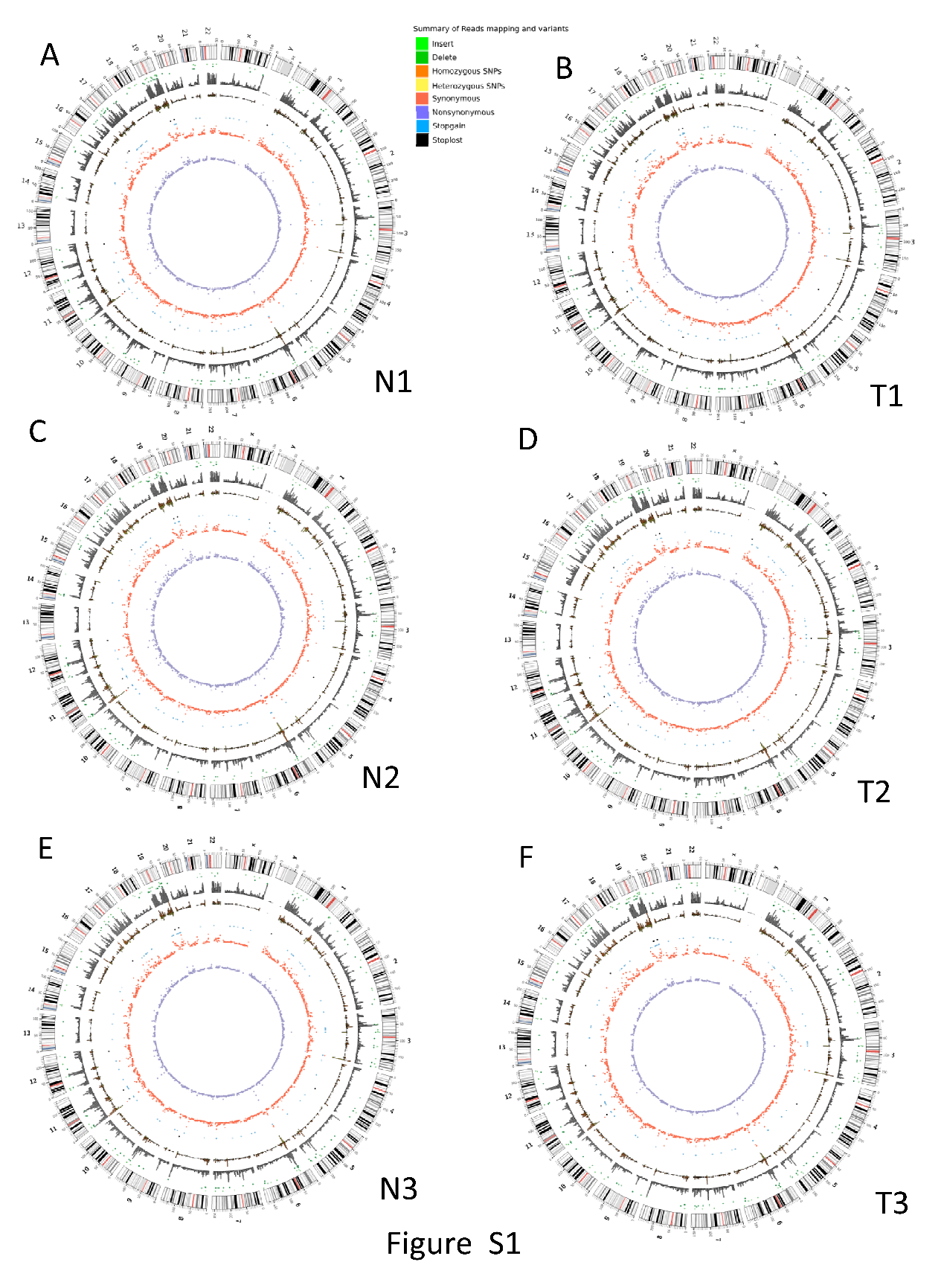
**

**Fig.S2**

**
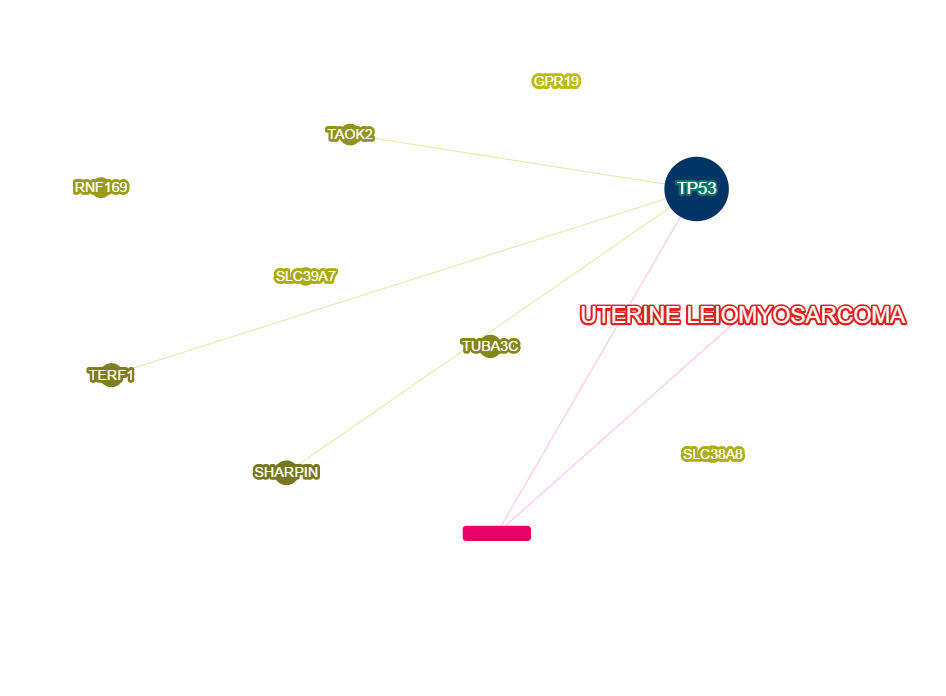
**

**Fig.S3a**

**
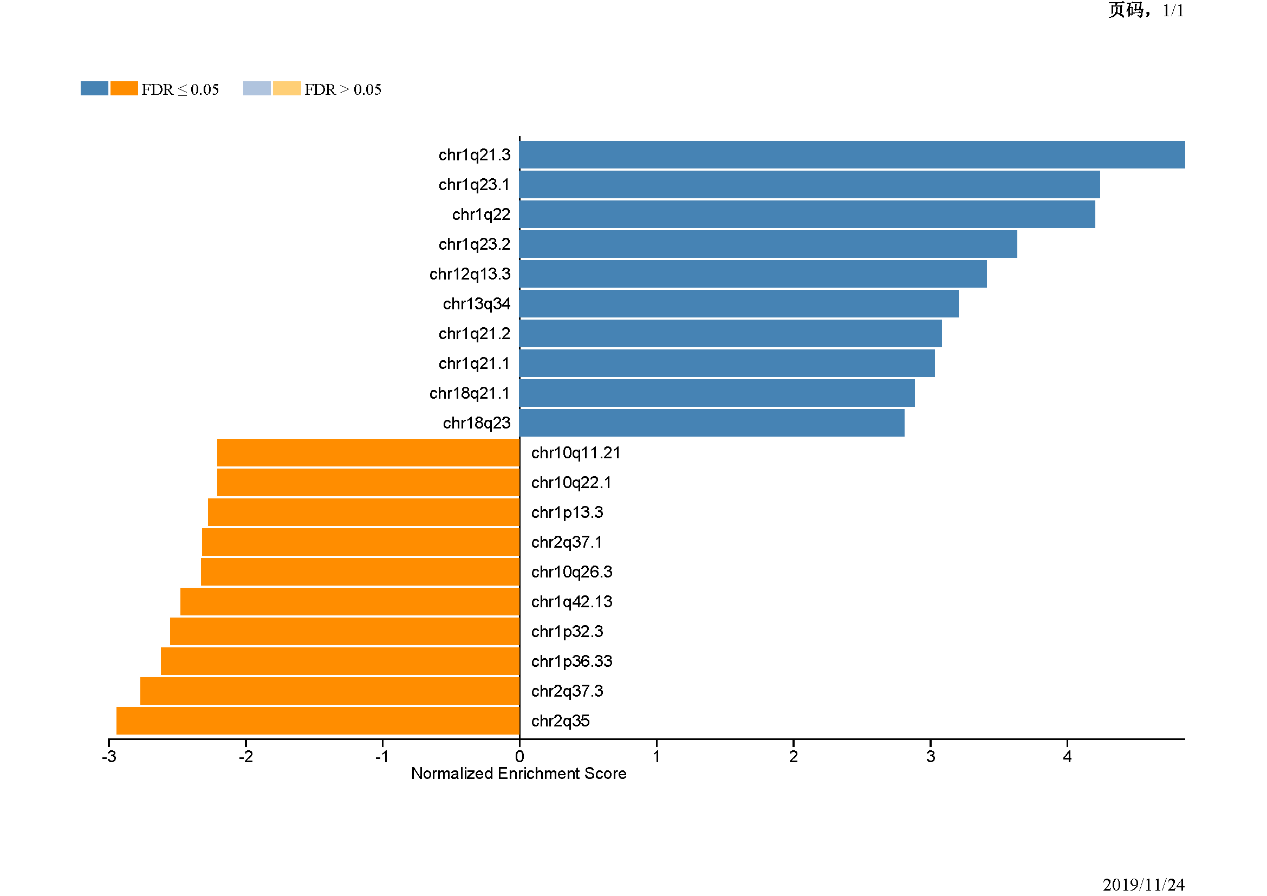
**

**Fig.S3b**

**
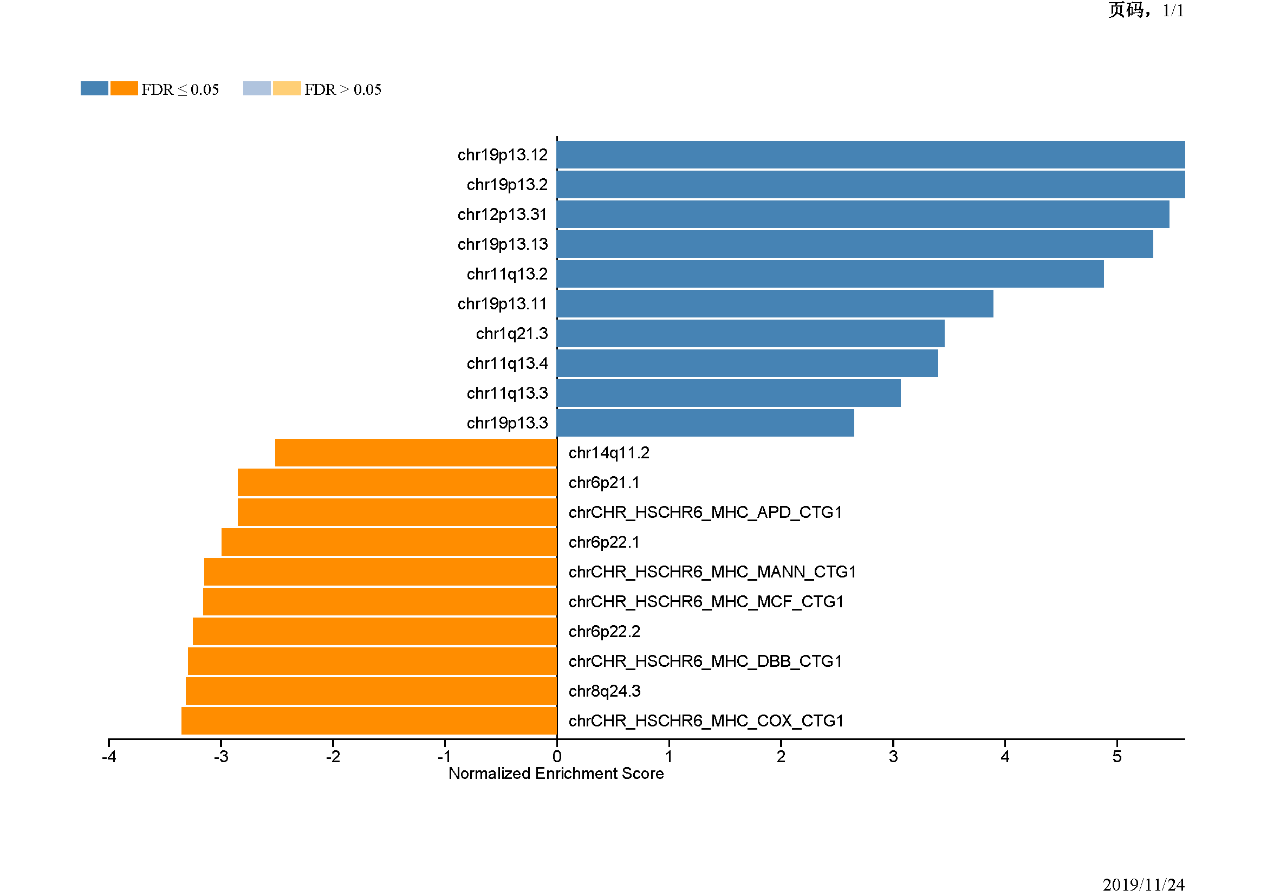
**

**Fig.S3c**

**
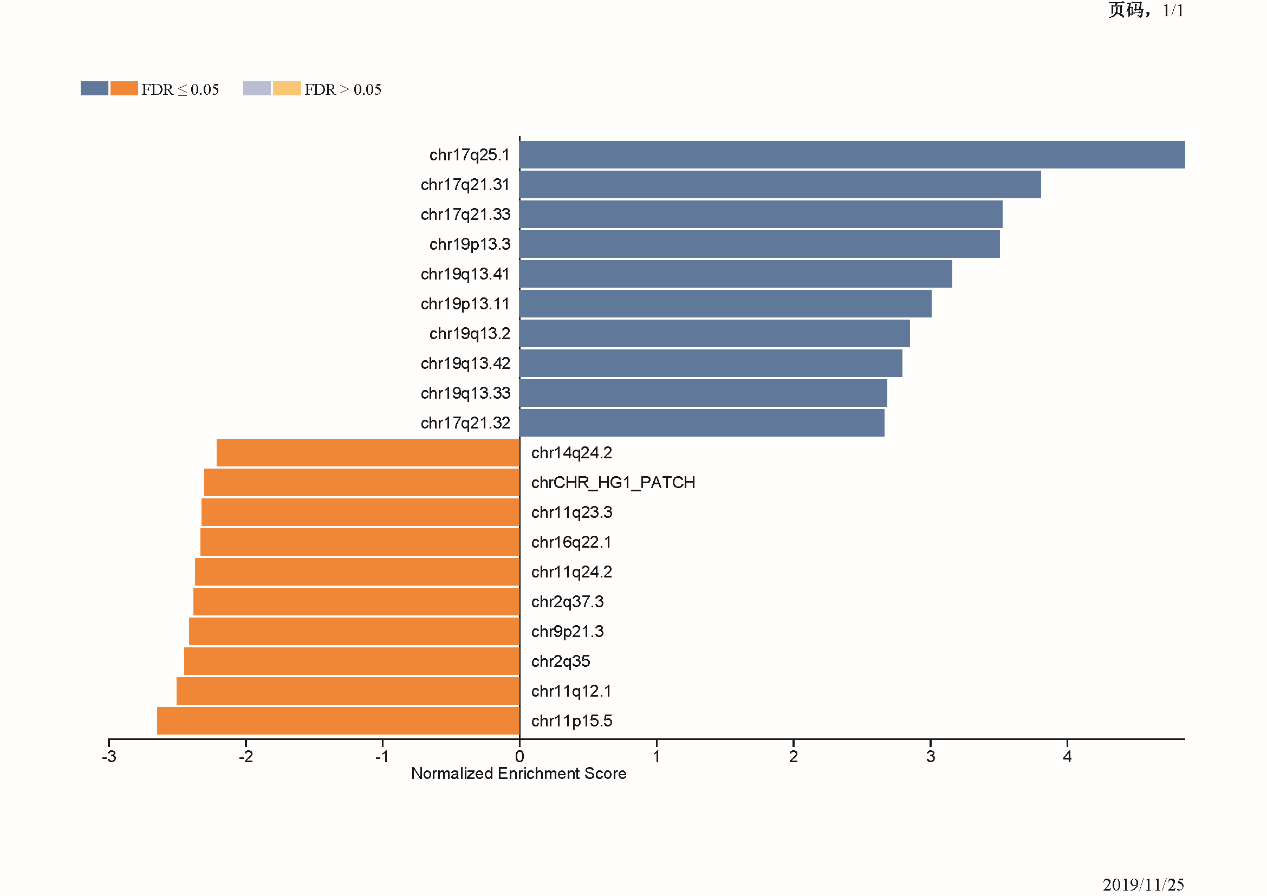
**

**Fig.S4a**

**
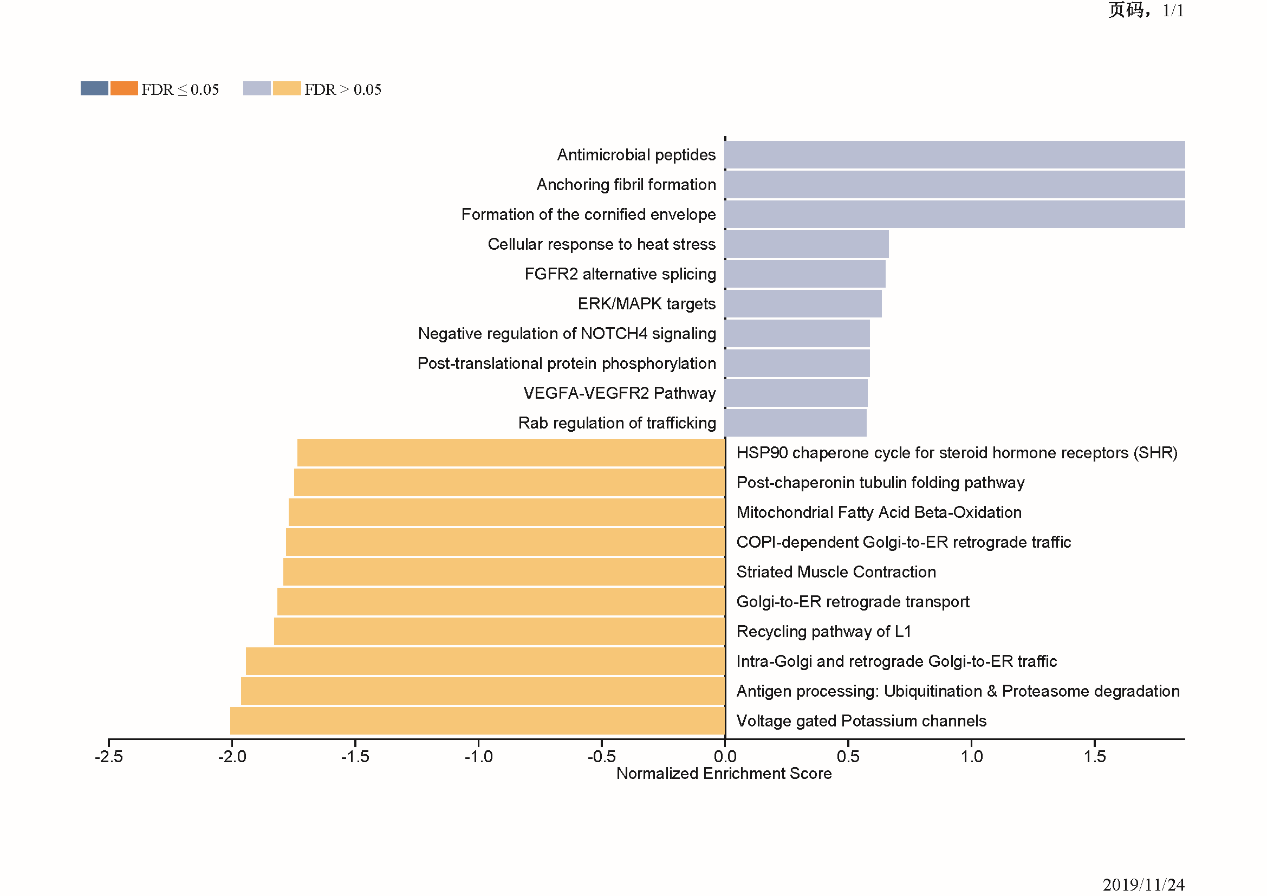
**

**Fig.S4b**

**
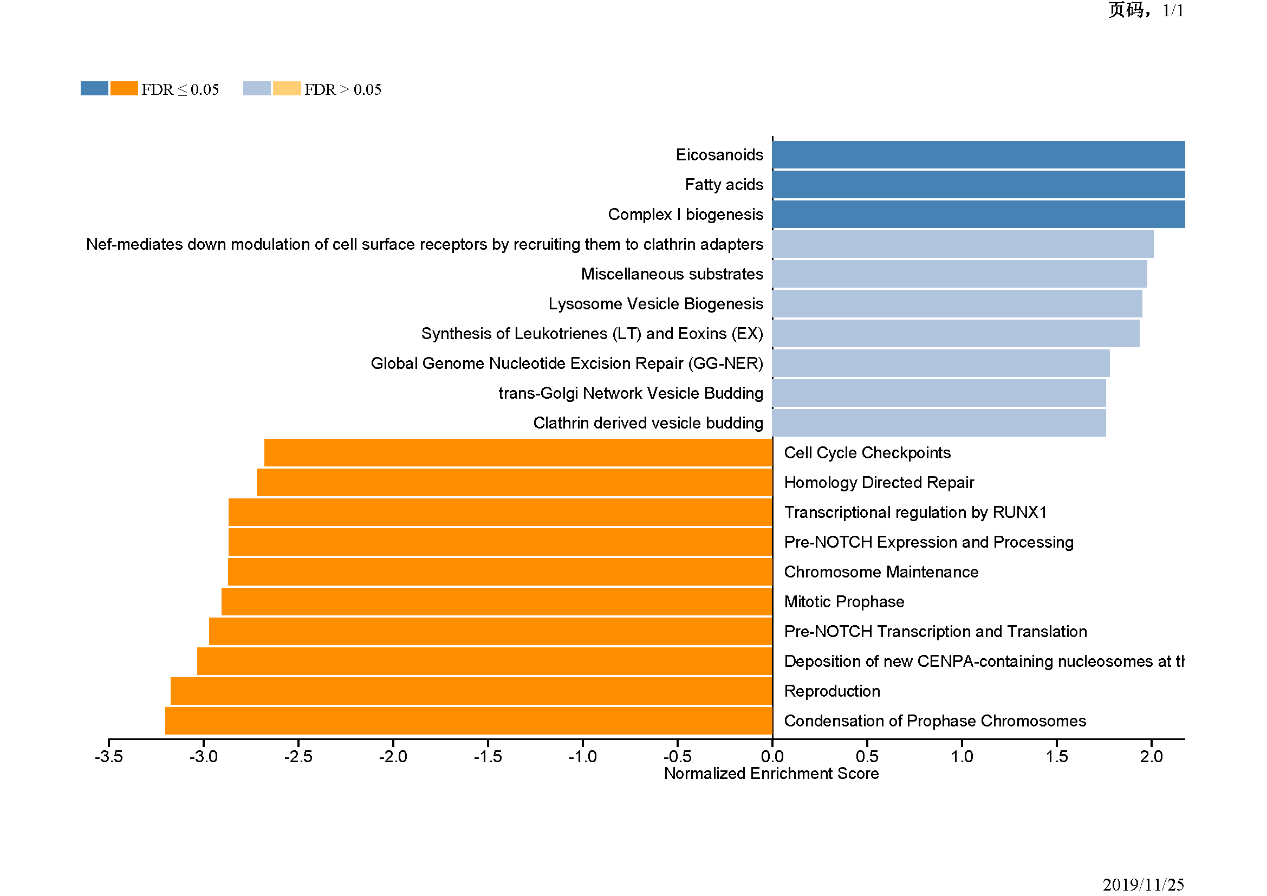
**

**Fig.S4c**

**
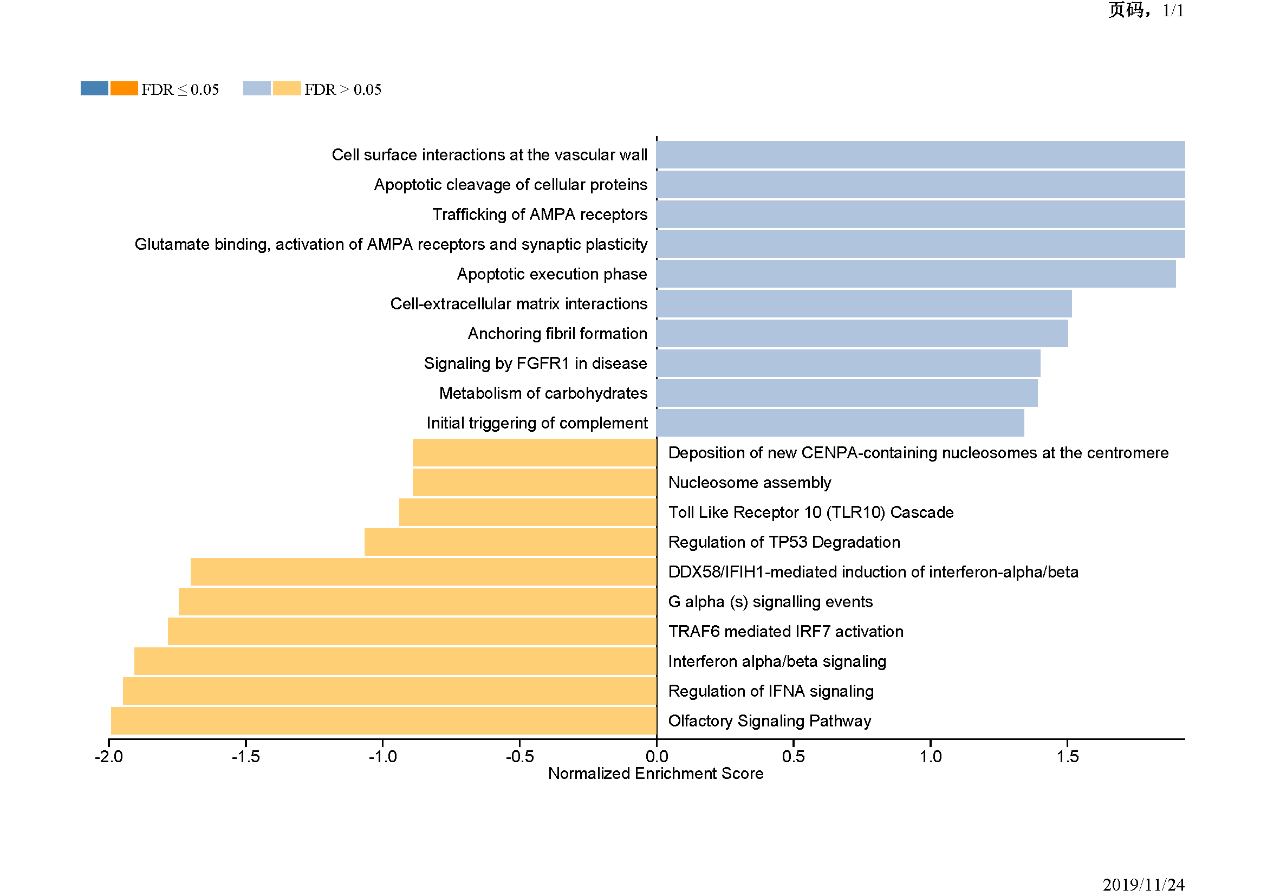
**

**Fig.S5**

**
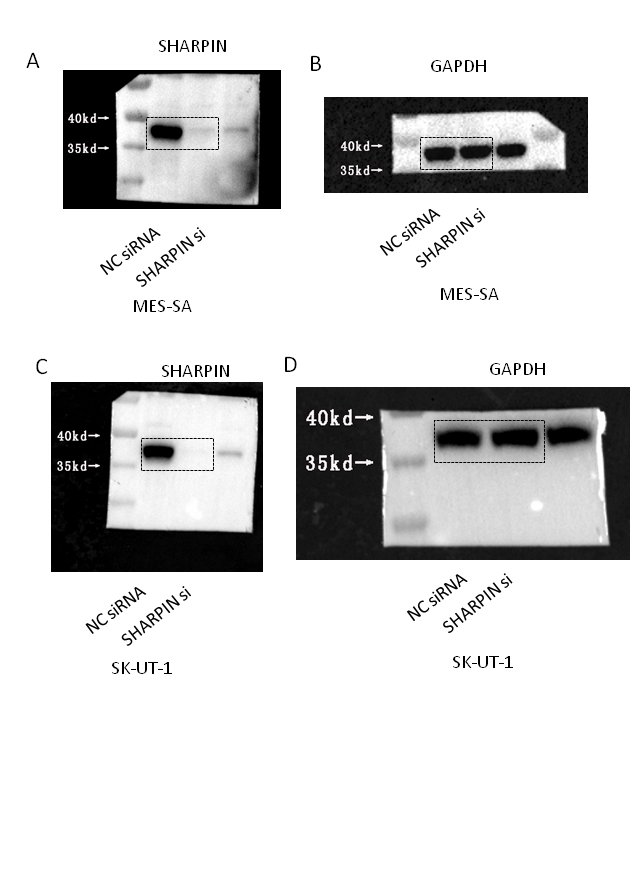
**
