# Supplementary material for: Identification of Somatic Genetic Alterations Using Whole-Exome Sequencing of Uterine Leiomyosarcoma Tumors
Source: Front Oncol. 2021 Jun 11;11:687899. doi: 10.3389/fonc.2021.687899 (PMC8226214; doi:10.3389/fonc.2021.687899)
Supplement: Supplementary file 2 [file Table_1.docx]

Table S1 primers for TP53 direct sequence

| **Exon** | **Forward** | **Reverse** |
| --- | --- | --- |
| **Exon4** | CGTTCTGGTAAGGACAAGGG | GGAATCCCAAAGTTCCAAAC |
| **Exon5-6** | CTTTATCTGTTCACTTGTGCCC | tcaaataagCAGCAGGAGAAAG |
| **Exon7** | CTGCTTGCCACAGGTCTCC | TGATGAGAGGTGGATGGGTAG |
| **Exon8-9** | GGGACAGGTAGGACCTGATTTC | GGCATTTTGAGTGTTAGACTGG |
| **Exon10** | aacttgaaccatcttttaactcagg | GGAATCCTATGGCTTTCCAAC |
| **Exon11** | GGAAAAGGGGCACAGACC | GCAGGGGAGGGAGAGATG |

Table S2. Depth and coverage of exomic sequence.

| **Exon Capture Statistics** | **N1** | **T1** | **N2** | **T 2** | **N3** | **T3** |
| --- | --- | --- | --- | --- | --- | --- |
| Target size(bp) | 62,085,295 | 62,085,295 | 62,085,295 | 62,085,295 | 62,085,295 | 62,085,295 |
| Clean reads | 94,595,974 | 98,204,792 | 100,704,888 | 78,417,370 | 62,858,796 | 88,963,148 |
| Aligned reads | 93,177,007 | 96,729,365 | 99,514,716 | 77,346,658 | 62,134,652 | 87,922,995 |
| %Aligned reads | 98.50 | 98.50 | 98.82 | 98.63 | 98.85 | 98.83 |
| Reads in target region | 53,565,386 | 55,069,778 | 56,101,629 | 44,252,215 | 35,106,870 | 50,493,503 |
| %Reads in target region | 57.49 | 56.93 | 56.38 | 57.21 | 56.50 | 57.43 |
| Mean depth of target region | 86.28 | 88.70 | 90.36 | 71.28 | 56.55 | 81.33 |
| Coverage of target region | 90.61 | 90.81 | 90.30 | 89.23 | 89.03 | 90.04 |
| Reads in flanking region | 29,680,252 | 29,941,234 | 31,538,047 | 24,795,886 | 19,529,947 | 28,197,970 |
| %Reads in flanking region | 31.85 | 30.95 | 31.69 | 32.06 | 31.43 | 32.07 |
| Mean depth of flanking region | 41.30 | 41.66 | 43.88 | 34.50 | 27.17 | 39.24 |
| Coverage of flanking region | 70.85 | 70.36 | 70.95 | 68.56 | 68.55 | 69.74 |
| %Targeted region covered at depths of at least 4X | 89.98 | 90.27 | 89.43 | 88.29 | 87.95 | 89.26 |
| %Targeted region covered at depths of at least 10X | 87.94 | 88.42 | 86.91 | 85.59 | 84.82 | 86.83 |
| %Targeted region covered at depths of at least 20X | 84.33 | 85.11 | 83.12 | 81.41 | 80.10 | 82.88 |
| %Flanking region covered at depths of at least 4X | 67.79 | 67.55 | 67.55 | 64.88 | 64.38 | 66.35 |
| %Flanking region covered at depths of at least 10X | 60.08 | 60.37 | 59.53 | 56.37 | 54.87 | 58.17 |
| %Flanking region covered at depths of at least 20X | 50.11 | 50.66 | 49.69 | 46.04 | 43.52 | 48.01 |
| Non-duplicated reads | 37,323,844 | 37,340,303 | 39,914,230 | 32,704,357 | 28,419,306 | 35,920,018 |
| %Non-duplicated reads | 40.06 | 38.60 | 40.11 | 42.28 | 45.74 | 40.85 |
| Uniq Mapped reads | 33,736,317 | 33,641,057 | 36,050,095 | 29,442,029 | 25,463,385 | 32,440,821 |
| % Uniq Mapped reads | 36.21 | 34.78 | 36.23 | 38.07 | 40.98 | 36.90 |

N: normal, T: tumor.

Table S3. Anotation of SNVs

| Categories | **Type of SNVs** | **N1** | **T1** | **N2** | **T2** | **N3** | **T3** |
| --- | --- | --- | --- | --- | --- | --- | --- |
| Total SNVs | Total SNVs | 184,428 | 175,692 | 184,924 | 161,148 | 155,459 | 174,660 |
| Whether known in database* | Known SNVs | 174,988 | 166,872 | 175,807 | 153,247 | 148,315 | 165,827 |
|  | Novel SNVs | 9,440 | 8,820 | 9,117 | 7,901 | 7,144 | 8,833 |
| Heterozygosity of SNVs | Homozygous | 88,439 | 86,207 | 87,735 | 80,425 | 73,048 | 89,574 |
|  | Heterozygous | 95,989 | 89,485 | 97,189 | 80,723 | 82,411 | 85,086 |
| Gene elements classifation annotation | downstream | 1,854 | 1,857 | 2,034 | 1,704 | 1,608 | 1,842 |
|  | exonic | 20,702 | 20,404 | 20,754 | 19,659 | 20,027 | 19,790 |
|  | exonic&splicing | 265 | 262 | 261 | 251 | 262 | 258 |
|  | intergenic | 60,690 | 56,261 | 59,227 | 48,805 | 45,449 | 56,130 |
|  | intronic | 68,514 | 65,157 | 69,486 | 60,787 | 58,169 | 65,600 |
|  | ncRNA | 10,326 | 9,971 | 10,822 | 9,673 | 9,347 | 10,046 |
|  | splicing | 87 | 95 | 83 | 79 | 85 | 87 |
|  | upstream | 2,794 | 2,771 | 2,851 | 2,403 | 2,448 | 2,813 |
|  | upstream&downstream | 127 | 125 | 137 | 120 | 124 | 128 |
|  | UTR3 | 16,101 | 15,867 | 16,330 | 14,924 | 15,197 | 15,105 |
|  | UTR5 | 2,965 | 2,919 | 2,937 | 2,741 | 2,739 | 2,857 |
|  | UTR5&UTR3 | 3 | 3 | 2 | 2 | 4 | 4 |
| Gene coding variation annotation | synonymous | 10,611 | 10,411 | 10,675 | 10,162 | 10,211 | 10,062 |
|  | nonsynonymous | 9,871 | 9,773 | 9,870 | 9,301 | 9,613 | 9,513 |
|  | stopgain | 77 | 82 | 85 | 80 | 84 | 86 |
|  | stoploss | 10 | 10 | 8 | 10 | 9 | 8 |

^*^Database:1000Genome(1000g2012apr)、dbSNP137、ESP6500.

Table S4. Exonic variant list from the wANNOVAR output after filtration.

| type | Gene variant | P value FDR |
| --- | --- | --- |
| nonsynonymous SNV | TERF1:NM_003218:exon1:c.C37G:p.R13G,TERF1:NM_017489:exon1:c.C37G:p.R13G, | <00001 |
| nonsynonymous SNV | TAOK2:NM_016151:exon3:c.C145T:p.R49W,TAOK2:NM_004783:exon3:c.C145T:p.R49W,TAOK2:NM_001252043:exon3:c.C145T:p.R49W, | . |
| nonsynonymous SNV | GPR19:NM_006143:exon4:c.C317G:p.S106C, |  |
| nonsynonymous SNV | TUBA3C:NM_006001:exon4:c.G644A:p.R215H, | 0.58 |
| nonsynonymous SNV | GDPD2:NM_001171192:exon3:c.C181T:p.L61F,GDPD2:NM_017711:exon3:c.C181T:p.L61F, | 0.19 |
| nonsynonymous SNV | SLC38A8:NM_001080442:exon5:c.T662A:p.V221D, |  |
| nonsynonymous SNV | C3orf20:NM_001184958:exon4:c.C148T:p.R50C,C3orf20:NM_001184957:exon4:c.C148T:p.R50C,C3orf20:NM_032137:exon4:c.C514T:p.R172C, |  |
| nonsynonymous SNV | RNF169:NM_001098638:exon6:c.C1232T:p.P411L, | 0.3 |
| nonsynonymous SNV | SHARPIN:NM_030974:exon2:c.G264C:p.E88D, | <0001 |
| stopgain | ADGRB1:NM_001702:exon24:c.C3496T:p.Q1166X, | 0.3 |
| nonsynonymous SNV | TP53:NM_001276699:exon3:c.C265T:p.R89W,TP53:NM_001126118:exon6:c.C625T:p.R209W,TP53:NM_001126112:exon7:c.C742T:p.R248W,TP53:NM_001276761:exon7:c.C625T:p.R209W,TP53:NM_001126115:exon3:c.C346T:p.R116W,TP53:NM_001126113:exon7:c.C742T:p.R248W,TP53:NM_001276697:exon3:c.C265T:p.R89W,TP53:NM_001276695:exon7:c.C625T:p.R209W,TP53:NM_001276760:exon7:c.C625T:p.R209W,TP53:NM_001126117:exon3:c.C346T:p.R116W,TP53:NM_001126114:exon7:c.C742T:p.R248W,TP53:NM_000546:exon7:c.C742T:p.R248W,TP53:NM_001276696:exon7:c.C625T:p.R209W,TP53:NM_001276698:exon3:c.C265T:p.R89W,TP53:NM_001126116:exon3:c.C346T:p.R116W, |  |
| nonsynonymous SNV | SLC39A7:NM_001288777:exon8:c.G1030C:p.E344Q,SLC39A7:NM_001077516:exon8:c.G1405C:p.E469Q,SLC39A7:NM_006979:exon7:c.G1405C:p.E469Q, | <0.0001 |
| nonsynonymous SNV | ODF3:NM_001286136:exon4:c.G380A:p.R127Q,ODF3:NM_053280:exon4:c.G380A:p.R127Q, | 0.08 |

Table S5. Anontation of Indels

| Categories | **Type of SNVs** | **N1** | **T1** | **N2** | **T2** | **N3** | **T3** |
| --- | --- | --- | --- | --- | --- | --- | --- |
| Total  Indels | Total  Indels | 7,536 | 7,440 | 8,008 | 6,785 | 6,252 | 7,134 |
| Whether known in database* | Known Indels | 6,456 | 6,368 | 6,782 | 5,824 | 5,399 | 6,032 |
|  | Novel Indels | 1,080 | 1,072 | 1,226 | 961 | 853 | 1,102 |
| Gene elements classifation annotation | downstream | 89 | 82 | 94 | 59 | 69 | 87 |
|  | exonic | 252 | 254 | 253 | 260 | 247 | 268 |
|  | exonic&splicing | 10 | 11 | 9 | 9 | 10 | 11 |
|  | intergenic | 1,837 | 1,806 | 1,923 | 1,574 | 1,420 | 1,727 |
|  | intronic | 3,205 | 3,059 | 3,437 | 2,901 | 2,614 | 2,999 |
|  | ncRNA | 428 | 468 | 482 | 414 | 382 | 423 |
|  | splicing | 37 | 39 | 40 | 39 | 34 | 43 |
|  | upstream | 140 | 148 | 152 | 120 | 108 | 132 |
|  | upstream&downstream | 7 | 10 | 9 | 5 | 3 | 7 |
|  | UTR3 | 1,353 | 1,368 | 1,420 | 1,235 | 1,210 | 1,256 |
|  | UTR5 | 178 | 195 | 189 | 169 | 155 | 181 |
| Gene coding variation annotation | frameshift deletion | 52 | 52 | 54 | 60 | 37 | 44 |
|  | frameshift insertion | 33 | 29 | 29 | 35 | 37 | 33 |
|  | nonframeshift deletion | 70 | 77 | 75 | 72 | 81 | 93 |
|  | nonframeshift insertion | 52 | 51 | 54 | 52 | 50 | 56 |
|  | nonframeshift substitution | 0 | 0 | 0 | 0 | 1 | 1 |
|  | stopgain | 2 | 2 | 1 | 0 | 2 | 3 |
